# Micro/nanodevices for assessment and treatment in stomatology and ophthalmology

**DOI:** 10.1038/s41378-021-00238-1

**Published:** 2021-01-29

**Authors:** An’an Sheng, Long Lin, Jia Zhu, Jian Zhuang, Jian Li, Lingqian Chang, Huanyu Cheng

**Affiliations:** 1 The Institute of Single Cell Engineering, Beijing Advanced Innovation Center for Biomedical Engineering; School of Biological Science and Medical Engineering, Beihang University, 100191 Beijing, China; 2 Department of Stomatology, Xiang’An Hospital of Xiamen University, 361100 Xiamen, China; 3 School of Stomatology, North China University of Science and Technology, 063210 Tangshan, China; 4 Institute of Plastic Machinery and Plastic Engineering, School of Mechanical and Electrical Engineering, Beijing University of Chemical Technology, 100029 Beijing, China; 5 Department of Engineering Science and Mechanics, Pennsylvania State University, University Park, PA 16802 USA; 6 School of Biomedical Engineering, Research and Engineering Center of Biomedical Materials, Anhui Medical University, 230032 Hefei, China

**Keywords:** Biosensors, Biosensors

## Abstract

Micro/nanodevices have been widely applied for the real-time monitoring of intracellular activities and the delivery of exogenous substances in the past few years. This review focuses on miniaturized micro/nanodevices for assessment and treatment in stomatology and ophthalmology. We first summarize the recent progress in this field by examining the available materials and fabrication techniques, device design principles, mechanisms, and biosafety aspects of micro/nanodevices. Following a discussion of biochemical sensing technology from the cellular level to the tissue level for disease assessment, we then summarize the use of microneedles and other micro/nanodevices in the treatment of oral and ocular diseases and conditions, including oral cancer, eye wrinkles, keratitis, and infections. Along with the identified key challenges, this review concludes with future directions as a small fraction of vast opportunities, calling for joint efforts between clinicians and engineers with diverse backgrounds to help facilitate the rapid development of this burgeoning field in stomatology and ophthalmology.

## Introduction

**Fig. 1:**
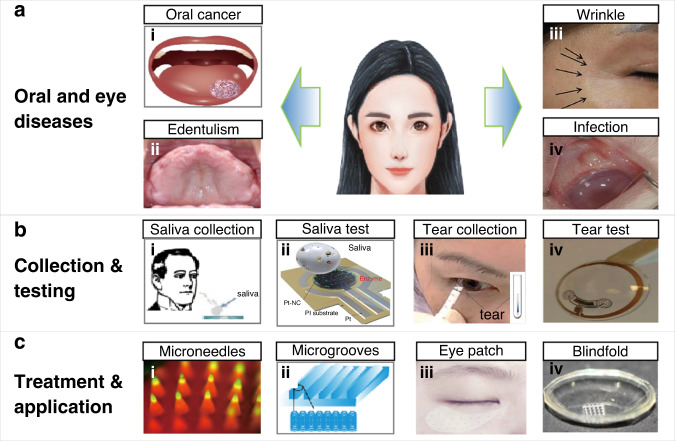
Micro/nanodevices for therapy and inspection in stomatology and ophthalmology. **a** Oral and eye diseases include oral cancer, edentulism, wrinkles, and infection. (i) Oral cancer manifests as red and white lesions in the early stage. As the disease progresses to an advanced stage, ulcers or exogenous tumors may appear^48^. (ii) Loss of dentition is the total loss of maxillary or mandibular teeth, which seriously affects chewing function, aesthetics, and speech. (iii) Wrinkles are obvious signs of skin aging, with structural changes around the orbit caused by internal factors (hormones and cellular metabolism) and external factors (long-term exposure, toxins, and ionizing radiation)^96^. (iv) Eye infections are caused by bacteria, microorganisms, and external factors^89^. **b** Biochemical sensing technology and micro/nanodevices can be used to detect markers in oral saliva and tears, analyze the detected substances, and provide support for the treatment of oral and eye diseases: (i) immunoelectrode for the detection of oral cancer; (ii) platinum nanocluster chemical sensor for measuring the hydrogen peroxide concentration^58^; (iii) measurement of the glucose concentration in tears for dry eye, glaucoma, keratitis, and other diseases caused by hyperglycemia^110^; and (iv) contact lens with integrated radio and sensor interface circuit for glucose detection^70^. **c** Micro/nanodevices can be used for assessment and treatment: (i) microneedle patch for the local administration of drugs to treat oral cancer^105^; (ii) microgrooves on the surface of the implant for antibacterial activity and mucosal sealing^82^; (iii) microneedles with drugs applied to the skin surface for eye wrinkle removal; and (iv) microneedle contact lenses to treat eye infections, such as keratitis^5^

In the past few decades, micro/nanodevices have emerged for the assessment and treatment of various diseases through the sampling of and drug release into cells and tissues of the mouth and eyes in stomatology and ophthalmology. From an anatomical point of view, the maxilla of the oral cavity, the suborbital area, and the eye are adjacent to each other. As a result, diseases that develop in one often influence the other. For instance, odontogenic infection of the maxillary teeth as an oral disease can cause local inflammation in the eye (e.g., acute retrobulbar optic neuritis and acute iridocyclitis)^1^. The close relationship is also reflected in the fact that both stomatology and ophthalmology fall into the same category of ophthalmology and otorhinolaryngology in the clinic. Furthermore, the biofluids (e.g., saliva and tears) secreted by glands in the mouth and eye exhibit overlapping microorganisms and biomarkers (e.g., proteins and enzymes), reflecting local oral/eye diseases, or systemic diseases^2^. Specific markers can be detected by simple, easy-to-use, sensitive, and highly reliable sensors and devices to indicate systemic symptoms. Similar topical medications are often used for the treatment of eye and oral diseases^3^. Oral cancer can be attributed to smoking, alcohol abuse, poor oral hygiene, and malnutrition, among others. Early oral cancer is asymptomatic and mainly manifests as red and white lesions. As the tumor grows larger, symptoms can include pain, numbness, repeated bleeding, and oral ulcers. Therefore, early diagnosis is critical for the prevention and timely treatment of oral cancer. Among common diseases of the oral cavity, edentulism affects chewing function and aesthetics and reduces quality of life. Ideally, complete dentures are implanted to restore function and appearance. In ophthalmology, dehydration and infection represent two common eye diseases^4^. The lack of water in the eyes in the former can cause dry eyes and sagging skin around the eyes. As the ability to retain moisture is compromised, the external manifestation often leads to wrinkles. Infectious eye diseases in the latter include keratitis, conjunctivitis retinitis, and blindness^5,6^. They can be caused by bacteria, viruses, or fungi. Efforts to address the above challenges have led to the rapid development of micro/nanodevices for the assessment, and treatment of oral and eye diseases (Fig. 1a). For instance, biochemical sensors have been developed to assist in the diagnosis of diseases through the sampling of enzymes and proteins of cells in saliva or tears (Fig. 1b)^7,8^. In particular, specific enzymes or proteins in oral cancer cells can be used to detect and continuously monitor tumor changes in stomatology (e.g., angiotensinase II as a representative enzyme in oral mucosal epithelial cells for the quick detection and investigation of the new coronavirus)^9^. Tissue fluids, such as oral saliva, could contain a variety of enzymes and proteins expressed by oral cancer cells, so they can be explored to monitor the development of cancer cells in real time as well. In the field of ophthalmology, the relationship between these trace substances and eye diseases is studied by extracting cytokeratin-19 (CK-19) and matrix metalloproteinase 1 (MMP-1) from cells. Meanwhile, the blood glucose level can also be determined from the tear glucose level.

As a simple yet effective treatment method, the transdermal drug delivery or even intracellular delivery of exogenous substances with micro/nanodevices has been extensively investigated. A wide variety of cargos, such as lidocaine (narcotic drugs), insulin, vaccines, besifloxacin (anti-inflammatory drugs), hyaluronic acid, and other drugs, are first preloaded into the devices. Next, they are delivered across the cell membrane or into tissue for anesthesia and vaccination or the treatment of diseases (e.g., diabetes, oral ulcers, wrinkles, and eye infections). In intracellular delivery, cellular membrane permeabilization realized by microinjection, optoporation, electroporation, and membrane penetration-mediated approaches can enable effective biochemical sensing and drug delivery^10–13^. As a painless and minimally invasive method^14^, transdermal drug delivery with microneedles can break through the stratum corneum or the surface of the oral mucosa to temporarily open microchannels, leading to the efficient and continuous treatment of oral mucosal diseases, and oral and maxillofacial tumor diseases^15,16^. These microneedle platforms are promising for the treatment of even diseases derived from genetic mutations and incorrect cell gene expression in the oral and maxillofacial regions^17^. Furthermore, microneedle eye patches and contact lenses are also used in clinical settings to treat eye wrinkles and eye infections. It should also be noted that surface plaque retention on oral implants can cause peri-implant inflammation and peri-implant mucositis. One potential solution is to explore photolithography-prepared microgrooves on the implant surface for improved antibacterial properties and biocompatibility, as well as an increased lifetime (Fig. 1c)^18^.

In this review, we present state-of-the-art developments and applications of micro/nanodevices in stomatology and ophthalmology. After introducing the challenges involved in the existing assessment and treatment methods, we summarize the biochemical sensing technology for disease detection, including cell sensors for intracellular proteins and enzymes, as well as tissue sensors for trace substances in tears and saliva, and their applications in systemic diseases. Next, we introduce micro/nanodevices for drug delivery to treat eye infections and oral mucosal diseases, along with their applications in dentition defects and eye wrinkles. We then discuss the opportunities for future developments of micro/nanodevices in stomatology and ophthalmology, calling for joint efforts from both clinicians and engineers with backgrounds in cell biology, micro/nanotechnology, chemistry, and mechanics to address the challenges in this burgeoning field.

## Existing challenges of common devices

**Fig. 2:**
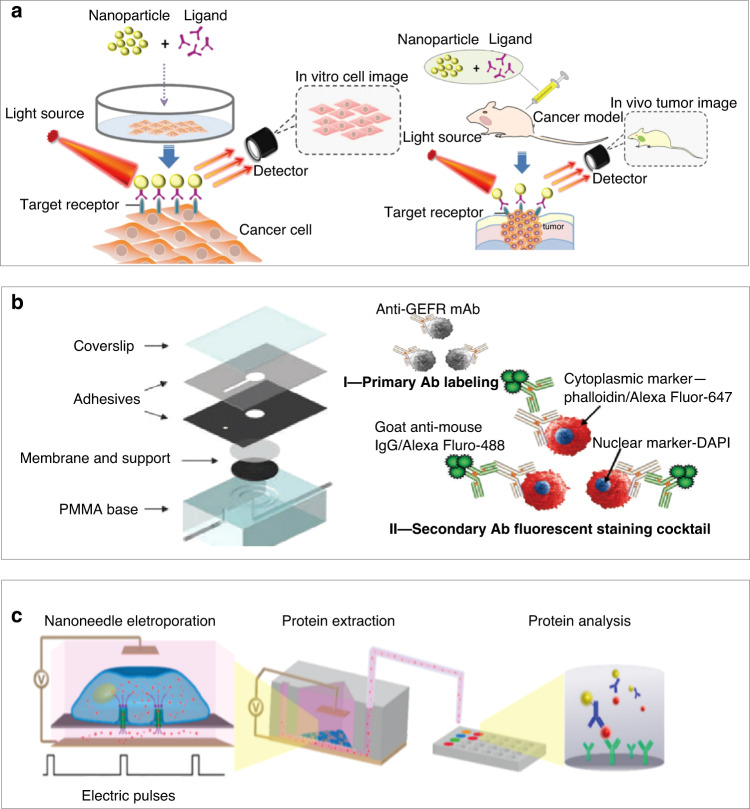
Novel biochemical cell sensors for stomatology. **a** Nanoparticles with a ligand can combine with a targeted receptor on cancer cells (e.g., SCC-25 cells), which can be imaged through a detection instrument under a light source, providing guidance for the treatment of oral cancer^48^. **b** The nanobiochip (NBC) sensor can capture oral squamous cells via this straightforward filtration mechanism on the membrane filter^49^. **c** Hollow nanoneedle electroporation systems for the nondestructive extraction of intracellular proteins (e.g., BCL-2 and lactate dehydrogenase B) from living cells into the microfluidic channel function by porating the cell membrane with electrical pulses; analysis is then performed with the standard enzyme-linked immunosorbent assay (ELISA)^12^

The anatomy of the oral cavity and eye is complex, so it is necessary to explore a variety of detection methods (e.g., imaging and biochemical methods), and surgical instruments to help with diagnosis and treatment in clinical settings (Fig. 2a, b). As representative imaging tools, X-ray radiography and computed tomography (CT) are used for the examination of auxiliary and deep tissues (e.g., salivary glands), as well as the detection of tumors^19^. Biochemical detection methods often require tears, saliva, and blood for qualitative and quantitative testing^20^. The application of surgical instruments (e.g., scalpel and electric knife) in the oral and maxillofacial regions, oral cavity, and eye for benign and malignant tumors often requires preoperative anesthesia, followed by surgical tumor removal.

### Oral diseases

In stomatology, the prevalent oral diseases include caries and pulp diseases, periapical periodontitis, periodontal disease, oral mucosal disease, and missing teeth^21^. Dental X-rays and CT scans are often used to assist in the diagnostic confirmation of pulpal and periapical diseases, along with preoperative examinations of oral implants. As an indispensable auxiliary diagnostic tool, biochemical detection, such as saliva testing, is often performed to screen for salivary gland diseases, such as Sjogren’s syndrome and burning mouth syndrome^22^. The quantitative detection of amylase in saliva with a detection reagent (α-amylase), and a colorimetric method can also be used to diagnose diabetes^23^. Following diagnostic confirmation, most diseases often require dentists to perform clinical operations, including dental pulp treatment, periodontal disease treatment, and dental implantation. Local anesthesia with injection needles or syringes is required to block the sensory conduction of nerve endings and fibers to reduce pain before invasive operations. While powerful, all of these tools are associated with limitations. For instance, radiation exposure is a common side effect of imaging examinations, making them unsuitable in certain patients, such as pregnant women and children^24^.

As a promising alternative, saliva and gingival crevicular fluid secreted from glands in the mouth can provide rich information for disease assessment^25^. These biofluids contain various proteins, microorganisms, and other components. The occurrence of certain diseases (e.g., periodontitis and oral cancer) can lead to changes in biomarker species and concentrations^26^. Therefore, the early detection of these biomarkers can objectively reflect the disease state for early diagnosis and treatment. For instance, inflammatory factors, such as interleukin (IL)-1β, IL-6, and MMP-8, can reflect the occurrence and development of periodontitis^27^. In the proteomic profile of oral cancer patients, elevation is observed in several biomarkers, including CYFRA-21-1, tissue polypeptide antigen, catalase, telomerase, α-amylase, albumin, glutathione, and proinflammatory factors. These proinflammatory factors include white blood cells, IL-6, IL-8, tumor necrosis factor-α, anti-P-53 antibody (Ab), and thioredoxin. Therefore, specific biomarkers can be used in early screening for oral cancer. The existing biochemical detection instruments have poor detection sensitivity and specificity.

### Eye diseases

Common diseases in ophthalmology include myopia, glaucoma, keratitis, xerophthalmia, and cataracts^28–30^. Cataract disease caused by aging and immunity and metabolic abnormalities, can be cured by surgery, but blindness caused by glaucoma is irreversible, as increasing pressure in the eye causes long-term compression of nerves and nerve atrophy^31^. Diabetic retinopathy is a disease caused by diabetes. Elevated blood glucose often causes systemic metabolic problems, crystal formation, and fluid turbidity, resulting in decreased light transmittance and then cataract disease^32^. At present, visual and instrumented inspections are still commonly used. For instance, fundus examination with an ophthalmoscope is required after pupil dilation to confirm myopia and determine the stage. Furthermore, handheld spectroscopically coded coherence tomography and reflection (SECTR) systems are commonly used for eye inspection^33^. The handheld probe uses a custom dual-pass scanning lens for full telecentric optical coherence tomography (OCT) scanning. However, these general inspection methods are not sufficient for the diagnostic confirmation of a specific disease, and they need to be combined with other inspection methods. This, biochemical detection methods are helpful. By considering the ratio of proteins, lipids, and small bioactive molecules in tears, the degree of corneal lesions can be confirmed and distinguished from bacterial corneal ulcers^34^. However, the insufficient calibration of existing testing equipment remains an unresolved issue in biochemical testing.

Topical medications are effective for treating infections, such as conjunctivitis and keratitis around the eye or on the surface of the eye, while they are less effective for treatments that require penetration of the cornea, such as retinal detachment repair and retinitis pigmentosa (RP) treatment. In addition to topical medication, surgical treatments may be needed for eye diseases affecting the vitreous body and retina. If external interventions are not effective (e.g., eye drops to treat ophthalmic fundus diseases, such as retinopathy), physicians may also perform injection therapy for invasive intravitreal, subretinal, and suprachoroidal administration^35^.

Similar to biofluids in the mouth, tears secreted from the ocular glands also contain essential biomarkers indicating the type of disease. For instance, the tears of patients with dry eye feature specific inflammatory factors, including calgranulin A and α-1 antitrypsin^36^, and an elevated transforming growth factor (TGF)-β1 level. Furthermore, lens protein expression in the retina is increased in retina-related diseases^37,38^.

The challenging issues and limitations of existing tools have spurred the rapid development of micro/nanodevices, including micro/nanobiochemical sensors and microneedle patches^6,39^. The former have high specificity and sensitivity and a short detection period, whereas the latter, with minimally invasive and painless features, allow for high-efficiency drug delivery^40^.

## Biochemical sensing technology

Biochemical sensors have been used to measure biomarkers in intercellular fluid, saliva, blood, tears, and sweat^41–45^. These biomarkers can be positively correlated with the occurrence of oral and eye diseases, which are also linked to systemic diseases. However, existing instruments, such as hematology analyzers and high-performance liquid chromatography systems, are bulky and require invasive blood sampling through a syringe. Efforts to address these challenges have led to the development of novel sensing micro/nanodevices (e.g., nanoneedles, electroporation systems, and immunoelectrode sensors) for the noninvasive, real-time monitoring of diseases through biomarker detection in cells and biofluids^46^.

### Micro/nanobiochemical sensors for oral disease in cells and tissues

With the integration of micro/nanodevices and the rapid advancement of fabrication technologies, devices targeting the cellular level are often safe, biocompatible, and noninvasive in stomatology^47^. Compared to current imaging contrast agents, nanoparticles with ligands exhibiting higher biocompatibility and easier synthesis processes can target-specific surface molecules (e.g., receptors on cancer cells due to ligand binding). By generating local surface plasmon resonance at near-infrared (NIR) wavelengths, nanoparticles provide higher image contrast and resolution. In particular, quantum dots, with high fluorescence intensity, low nonspecific binding, and good stability against optical drift, can be used for the real-time detection of SCC-25 oral squamous carcinoma cells to provide a basis for the targeted therapy of oral cancer patients (Fig. 2a)^48^. In particular, real-time observation of the epithelial layer and basement membrane of the detected cells (e.g., oral squamous cells, carcinoma cells, and abnormal hyperplasia cells) through OCT can enable the diagnosis of precancerous lesions and early oral cancer. Compared to biopsy, OCT with a high spatial resolution of ~10 μm can reduce pain and trauma. Capable of analyzing biomarkers along with biochemical and morphological changes in cytology specimens exfoliated from oral tumors, nanobiochip sensor technology can be used for the early detection of oral cancer. The process includes capturing cells on a membrane filter, followed by immunolabeling and staining with epidermal growth factor receptor (EGFR)/Alexa Fluor-488, phalloidin/Alexa Fluor-647, and DAPI for fluorescence imaging and analysis (Fig. 2b)^49^.

The detection of intracellular enzyme activity in vitro also plays a vital role in the diagnosis and detection of oral diseases. For instance, as the main host cell receptor for COVID-19, angiotensinase II (ACE-II) mediates the process by which the virus enters the cell to cause infection^50^. The composition and ratio of ACE-II expression in the oral cavity based on public RNA-seq profiles and single-cell transcriptomes also indicates that the expression of the COVID-19 receptor ACE-II receptor exists in the oral mucosa^9^. As another example, caspase-3 activation-mediated neuronal death is a common process in neurodegenerative diseases^51^. Nanodevices targeting the brain to apply fluorescence resonance energy transfer (FRET) induced by QSY-21 and CY5 can detect caspase-3 activated in apoptotic neurons. After the nanodevice enters the cell through endocytosis, intracellularly activated caspase-3 can lyse a specific sequence (DEVD), causing CY5 to detach (with a specific gene segment cleaved by ACE-II) and display fluorescence. FRET can also be extended to the analysis and detection of oral mucosal epithelial cells, with the use of nanoparticles to identify and penetrate the cell membrane, followed by the detection of ACE-II in oral mucosal epithelial cells by receptor-mediated endocytosis. Furthermore, a hollow nanoneedle electroporation system can be applied to screen patients with suspected tumors and in high-risk groups. Because small nanostraws with a diameter of 150 nm coupled with transient electric pulses are able to extract proteins, a large hollow nanoneedle with a diameter of ~450 nm can result in a higher protein diffusion rate in the nanoneedle channel. By combining nanoneedles with long-lasting electrical pulses, the extraction time can be extended for real-time monitoring. After applying the electrical pulse, intracellular proteins (e.g., BCL-2 as a biomarker for oral squamous cell carcinoma) from blood samples diffuse through the hollow nanoneedle into the microfluidic channel located beneath the cell, which can enable the noninvasive and real-time monitoring of tumor development (Fig. 2c)^12^. Although research efforts on the capture and extraction of live cells in stomatology do not exist at present, live cell monitoring methods can be applied for specific markers in oral squamous cell carcinoma cells (BCL-2) for the rapid detection of oral diseases in the future (Tables 1–3).

**Table 1:** Advantages and disadvantages of common and micro/nanodevices in stomatology and ophthalmology

	Stomatology	Ophthalmology
*Traditional devices*		
Advantages	Dental X-ray: auxiliary diagnosis, convenient and quick Saliva test: low cost, fast detection results Syringe: commercialized, can be produced in large quantities, and can be used quickly and conveniently	Ophthalmoscope: easy detection of common ocular problems, such as fundus lesions SECTR probe: wide application range, can be used to check cornea, aqueous humor, lens, optic disc, retina, choroid, etc. Eye drop: simple, easy, effective local treatment Mechanical drilling: direct drug delivery into the retina
Challenges	Dental X-ray: needs to be combined with clinical examination Saliva test: poor detection specificity due to oral cavity microorganisms and other interference factors Syringe: pain	Ophthalmoscope: special problems, such as abnormal intraocular pressure, cannot be detected Eye drop: poor permeability, only suitable for local surface inflammation and infection Mechanical drilling: technically sensitive, traumatic
*Micro/nanodevices*		
Advantages	Saliva immune sensor: good specificity and sensitivity, can eliminate interference Microneedle patch: minimally invasive, painless, and highly effective drug delivery	Tear sensor: easy to extract sample, simple to operate, specific detection of biomarkers Eye patch, contact lens: local, painless, minimally invasive, efficient administration
Challenges	Saliva immune sensor: expensive, currently in the research phase Microneedle patch: currently, the dose and rate of drug release need to be customized	Tear sensor: detection of many interference factors (inorganic salt, protein, external impurities, etc.) Eye patch, contact lens: independent testing and treatment, imperfect system

**Table 2:** Performance comparison of biosensors in stomatology and ophthalmology

Materials	Key process	Biomarker/testing sample	Electrode/substrate	Range	Sensitivity	Ref.
Biofunctionalized nanostructured zirconia	Electrophoretic deposition, drop-casting method	Saliva/CYFRA-21-1	ITO	2–16 ng mL^−1^	2.2 mA mL ng^−1^	^8^
Mouthguard glucose sensor	Electrochemical process	Saliva/glucose	Pt Ag/AgCl	20–200 μM	/	^54^
Enzyme-based electrochemical biosensor	Spin-coating	Saliva/cholesterol	Pt or glassy carbon	2–486 μM	132 μA mM^−1^ cm^−2^	^58^
Metabolite biosensor	Two-step dip-coating	Saliva/glucose/lactate	Pt	/	10^−7^ M/10^−6^ M	^56^
Wearable contact lens biosensor	Polymer drop-casting	Tear/glucose	PS	0–50 mM	12 nm mM^−1^	^69^
Soft contact lens biosensor	Enzyme immobilization	Tear/glucose	Polydimethylsiloxane	0.03–5.0 mmol L^−1^	/	^64^

**Table 3:** Performance comparison of micro/nanodevices for treatment in stomatology and ophthalmology

Device	Material	Key process	Working principle	Feature	Ref.
Nanoantibiotic platform	Gold nanocage, poly(N-isopropylacrylamide-co-diethylaminoethyl methacrylate) (PND)	Melting	NIR-induced release of tetracycline	On-demand release	^74^
Microneedle	PAM-PDA hydrogel	Micromolding	Transdermal drug delivery	Hydrogel + microneedle method, reduced pain, higher anesthetic accuracy, and faster recovery	^78^
Nano- and microstructures with nanoscale hydroxyapatite	CaTiO_3_	Microarc oxidation (MAO)	Oral implant surface modification to promote osteoblast proliferation and osteogenic differentiation for osseointegration in bone repair	Nano/microhierarchical structure promoted cell proliferation and osteogenic differentiation	^81^
TiO_2_ nanopores	TiO_2_	Electrochemical anodization	Nanopores in oral implant modulate specific cell proliferation and alignment	Nanopore structure is compact and dense for enhanced mechanical strength	^18^
Thermoresponsive microneedles	Graphene, gold	Chemical vapor deposition	Microneedle patch can be thermally actuated to deliver metformin	Temperature, humidity, glucose and pH sensors, and polymeric transcutaneous delivery	^6^
Layered double hydroxide nanoparticle contact lens	Layered double hydroxide PLGA-PEG	Copolymerization	Thermoresponsive drug release	Drug release from special contact lens made of Bri@LDH/Thermogel	^88^

**Fig. 3:**
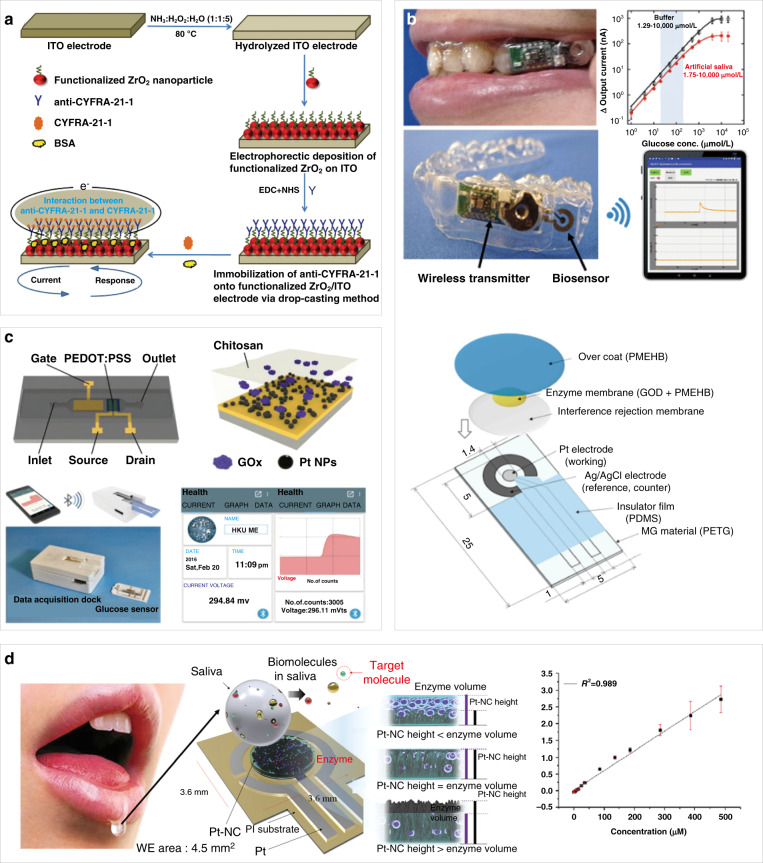
Biochemical sensors for biomarker detection in saliva. **a** Fabrication of immune electrodes for saliva-based oral cancer detection^8^. **b** A mouthguard sensor integrated with a wireless module and battery; the schematic (bottom) shows that the glucose sensor consists of an anti-interference membrane and an enzyme membrane on Pt and Ag/AgCl electrodes^54^. **c** Organic electrochemical transistor (OECT) based on platinum nanoparticles modified with glucose oxidase and polyvinyl alcohol (PVA) for a highly sensitive, portable glucose biosensor^56^. **d** Determination of cholesterol in saliva by platinum nanocluster chemical sensors. The device catalyzes the reaction of cholesterol in saliva by cholesterol enzymes to produce hydrogen peroxide and measures the concentration of cholesterol by measuring the concentration of hydrogen peroxide^58^. Calibration curve of the Pt-NC/enzyme/Nafion sensor showing the current response to the continuous addition of total cholesterol under continuous stirring in 0.1 M PBS buffer^58^

The proteins and enzymes in biofluids can also be detected by micro/nanobiomedical devices for the diagnosis of oral diseases^52,53^. In addition to moistening the mouth and protecting the oral mucosa, the saliva generated from three major salivary glands (parotid gland, submandibular gland, and sublingual gland) also contains specific proteins useful for diagnosing cancer of the oral cavity. Although the currently used biomarkers for oral cancer detection include IL-8, IL-6, vascular endothelial growth factor, and EGFR, their concentrations are low in saliva. In contrast, the high concentration of CYFRA-21-1 in saliva makes it easy to use as a biomarker for oral cancer; additionally, its level is four to five times higher in the saliva of oral cancer patients than in that of healthy individuals. Coating biocompatible, nanostructured zirconium oxide with organic functional anti-CYFRA-21-1 molecules in an oral cancer immunoelectrode sensor allows it to bind with the biomarker protein CYFRA-21-1, which produces oxidation current in the sensor to detect oral cancer (Fig. 3a)^8^. The immunoelectrode sensor exhibits a detection threshold of 2–16 ng mL^−1^ and a detection current of 0.176 mA with a 0.99% decrease in the oxidation current. The sensor is also highly selective because its oxidation current only decreases by 0.14%, 1.3%, 1.68%, and 1.75% upon the addition of carcinoembryonic antigen, carboxymethyl cellulose sodium, troponin I, and glucose, respectively.

In addition to cancer biomarkers, glucose and lactate in saliva can also be selectively detected to indicate the presence of a variety of oral diseases. The concentration of glucose in saliva is directly related to the concentration of glucose in blood. Although there have been many reports on saliva collection and sampling to detect glucose in saliva, research on the direct real-time sampling and analysis of saliva is still rare. Takahiro et al. designed a glucose sensor mouthguard to analyze saliva, with a layer of cellulose acetate film wrapped on the surface to eliminate the interference of ascorbic acid (AA) and uric acid (UA) in saliva (Fig. 3b)^54^. The working electrode and counting/reference electrode (RE) of the glucose sensor consisted of a 200-nm thick Pt electrode (1.6 mm^2^ in size) and a 300-nm thick Ag/AgCl electrode (16 mm^2^ in size). The other parts of the electrode included polydimethylsiloxane (PDMS) as a cover and insulator and poly(MPC-co-EHMA-co-MBP), with excellent biocompatibility and high mechanical strength, for the immobilization of glucose oxidase. This sensor can evaluate the glucose concentration in the range of 1.75–10000 mol L^−1^ and can be connected to a mobile phone for output monitoring. This is the first study to directly monitor the glucose concentration of saliva in the oral cavity without pretreatment. This approach could serve as a high-efficiency, noninvasive glucose detection method for diabetes.

As an example, in periodontal diseases, elevated glucose causes abnormal leukocyte chemotaxis and defects in phagocytosis, and thickens the vascular basement membrane, which reduces the anti-infective ability of periodontal tissues. The risk of caries and periodontal tissue also increases with increases in the blood glucose level, which directly correlates with the glucose level in saliva^55^. This is because high glucose levels in saliva lead to the formation of excessive lactic acid in the plaque metabolism, increasing the probability of caries and periodontal tissue infection. The highly selective detection of glucose and lactate in saliva can be achieved by organic electrochemical transistors with glucose oxidase/lactate oxidase- and poly(n-vinyl-2-pyrrolidone)-coated platinum nanoparticles (Pt NPs) on the gate^56^. After a two-step dip-coating process to deposit Pt NPs on the gate electrode, UV–ozone posttreatment is carried out to improve the catalytic ability of the Pt NPs. A double-layered membrane consisting of polyaniline (PANI) and perfluorosulfonic acid Nafion–graphene is then coated before decorating the surface with the outermost enzyme layer to result in a sodium fluoride-modified saliva glucose sensor. The coating of a double-layered membrane combining PANI and perfluorosulfonic acid (Nafion)–graphene between the outermost enzyme layer and the gate electrode helps prevent interference from other substances in saliva, including dopamine, UA, and AA. At the same time, the double-layered membrane can help fix glucose oxidase on the grid electrode to improve the glucose test performance. In addition to the improved sensitivity to glucose and lactic acid, the demonstrated detection limit is 10^−7^ M for glucose and 10^−6^ M for lactic acid (Fig. 3c)^56^. The glucose level in human saliva samples was measured to increase from 4.2 × 10^−5^ to 1 × 10^−4^, which is in good agreement with the normal saliva glucose concentration of healthy people. The detection limit for lactate is slightly higher than that for glucose, but it is sufficient to measure the lactate concentration range.

It is also important to measure cholesterol levels because high cholesterol results in risks for hypertension and myocardial infarction that are positively correlated with the occurrence of periodontitis. Higher cholesterol levels can also accelerate alveolar bone resorption and the progression of periodontitis^57^. The immobilization of a cholesterol enzyme on the optimized vertical structure of a platinum nanocluster (Pt-NC) results in a noninvasive electrochemical biosensor to rapidly and accurately detect salivary cholesterol (Fig. 3d)^58^, the level of which also correlates with the cholesterol concentration in the blood. The biosensor exhibits a linear range from 2 to 486 μM, with a sensitivity of 132 μA mM^−1^ cm^−2^.

### Micro/nanobiochemical sensors for eye disease at the cell and tissue levels

**Fig. 4:**
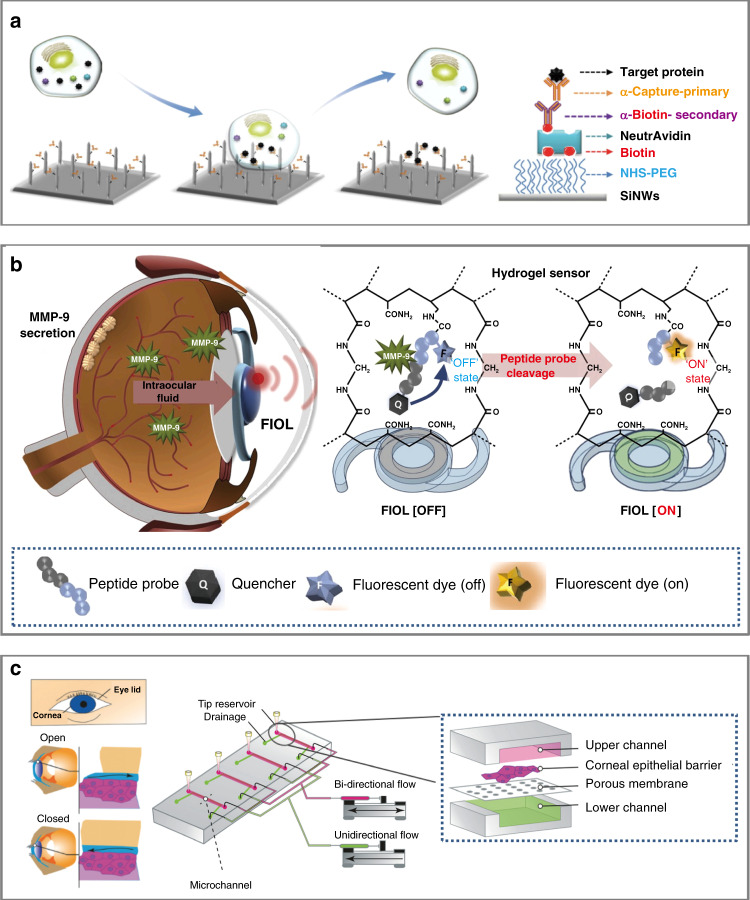
Biosensors for eye detection at the single-cell level. **a** Antibody-modified vertical silicon nanowire array (VA-NW) for capturing intracellular proteins from living cells for in situ and in vitro intracellular delivery and sampling^59^. **b** Fluorogenic intraocular lens (FIOL) for the in vivo monitoring of MMP-9, a target in the aqueous humor (AH), to diagnose and monitor chronic progressive diseases, such as glaucoma. The fluorescent peptide probe in the fluorescence resonance energy transfer (FRET)-OFF state selectively cleaved by MMP-9 changes to the FRET-ON state for an enhanced fluorescence signal^61^. **c** (Left) dynamic movement of the tear film during blinking on the human cornea. (center) Schematic diagram of a dynamic blood perfusion system. The upper channel is connected to a syringe pump that produces bidirectional flow, while the lower channel is connected to another syringe pump that produces unidirectional flow. The micropipette tip is fixed in the outlet of the upper chamber and used as a storage tank. During blinking, tears continuously produce shear stress on the corneal epithelium^63^

Capable of manipulating cells in vivo, in situ, and in vitro, the vertical nanowire (VA-NW) array is powerful for detecting intracellular biomarkers and delivering biologically active substances directly into cells. In combination with a microfluidic device, the VA-NW array can promote intracellular delivery and sampling by penetrating cell membranes for membrane disruption. Nondestructive sampling with solid NWs eliminates the need for cell lysis, which is an improvement over traditional protein pull-down methods. In a typical process, proteins are captured using target-specific Abs immobilized on the NW surface. The surface of the VA-NW is covered with a polyethylene glycol layer to prevent strong cell adhesion and improve the rate of cell recovery. Suspended K562 human chronic myelogenous leukemia cells transiently transfected to express red fluorescent protein (RFP) were seeded on anti-RFP Ab-functionalized VA-SiNW arrays and incubated for 24 h. The cells were washed off the NWs and remained viable, with no adverse effect on membrane integrity. The fluorescence of the NWs indicated the successful pull-down of the protein (Fig. 4a)^59^. These results indicate that the multifunctional protein cluster is the most highly expressed gene transcript in the human cornea. While the protein mainly exists in the cornea and conjunctival epithelium, it also appears in human tears. The multifunctional protein acts as a barrier to seal the ocular surface and protect the ocular surface from drying, when its concentration is higher than a threshold value. This finding may become a foothold for investigating new clinical treatments for dry eye in ophthalmology^60^.

As an extension of the central nervous system (CNS), the eye contains aqueous humor (AH), and the fluid secreted from tissues in the eye is rich in biomolecules. The detection of enzymes (e.g., MMP-9 and MMP-1) and proteins (cluster) in cells/tissues and tears with micro/nanobiomedical devices is directly relevant for eye diseases (i.e., keratitis, keratoconus (KC) disease, and xerophthalmia). Therefore, collecting and analyzing easily accessible biomarkers in AH (e.g., MMP-9, MMP-1, and TUBA) for the diagnosis of ophthalmological diseases and early changes in CNS diseases is of significant interest. As an emerging implantable device for intraocular detection, the intraocular lens (IOL) sensing system detects AH through a fluorescent hydrogel sensor activated by biocompatible enzymes and biomarkers. The demonstration of this sensor in rabbit eyes involved its use to detect metal-based protease (MMP-9), one of the biomarkers of CNS and ocular diseases. By implanting fluorogenic IOL (FIOL) into the eyes of rabbits with cataracts, the hydrogel sensor with peptide probes can be used to relate the fluorescence signal (observed with a slit lamp under cobalt blue-filtered light) to the increase in the concentration of MMP-9 in rabbit corneal endothelial cells. Because of the low levels of clotting factors and mixed solubility in AH, implantation of the new semipermanent FIOL device inside the eye allows for the continuous detection of ocular biomarker levels (e.g., glucose concentration and intraocular pressure (IOP); Fig. 4b)^61^, which could serve as a basis for using tear fluid in clinical IOL-based liquid biopsy. Because some glaucoma patients have IOP values in the normal range of 12–22 mmHg, ophthalmoscopic examination results can be inaccurate. Efforts to address this challenge have led to the possible use of MMP-9 detection by a fluorescence sensor for more accurate diagnosis and monitoring^61^.

Some genes (VSX1, TGFBI, and TUBA3D) can also cause degenerative corneal disease, such as TUBA3D in KC disease, a dilatation, thinning, and conical projection of the cornea that can lead to impaired vision, irregular astigmatism, and corneal scarring. The mutant protein of TUBA3D is unstable and may lead to increased MMP expression and oxidative stress in human corneal fibroblasts, thereby reducing the extracellular matrix within the cornea, and resulting in stromal thinning and damage to the Bowman layer/basement membrane (characteristic of KC)^62^. In addition to molecular markers, the dynamic monitoring of eye blinking can be helpful because it not only affects the secretion of tears, but also hinders the evaluation of corneal function. Consisting of upper and lower channels separated by a porous membrane, a microfluidic platform was developed to dynamically cultivate the human corneal barrier to reproduce blinking. After seeding HCE-T human corneal epithelial cells on the porous membrane (upper channel), two-way and one-way flow was applied to the upper and lower channel, respectively, to stimulate the cells in the upper channel with a shear force. After 24 h, the significantly increased intracellular CK-19 intermediate filament expression supported and assisted the stretching process of the initially flat cells, indicating enhanced barrier function. The established model can help evaluate the integrity of HCE-T cells, perform ophthalmic drug permeability and toxicity tests, and identify methods to overcome the corneal barrier and blinking factors in clinical practice (Fig. 4c)^63^.

**Fig. 5:**
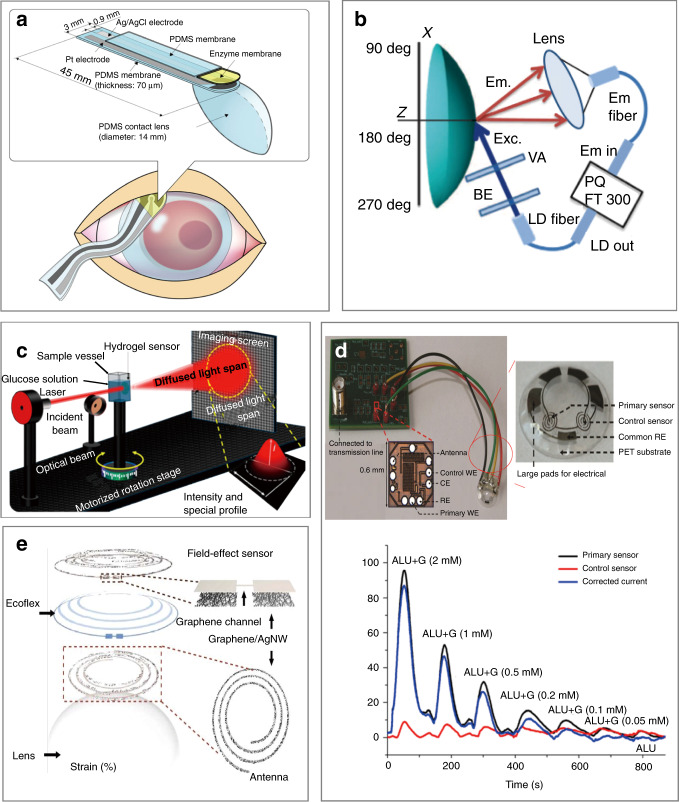
Biochemical sensors for tear fluid analysis. **a** Soft contact lens (SCL) biosensor worn on the eye to monitor tears in situ^64^. **b** Optical setup to measure the fluorescence on the surface of the contact lens. BE beam expander, VA variable aperture, LD laser diode^7^. **c** Monochromatic light transmission device based on a hydrogel sensor to measure the glucose concentration according to optical responses^68^. **d** Integrated contact lens with a differential enzymatic glucose sensor and communication circuit^70^. Typical continuous responses from test solutions at different glucose concentrations and interfering ALU. Tear fluid ALU: ascorbic acid 50 μM, lactate 10 mM, and urea 10 mM (ref. ^70^). **e** Schematic diagram of a multifunctional wearable contact lens composed of a glucose sensor and an intraocular pressure sensor^71^

Secreted from the lacrimal gland, tear fluid includes various proteins and lysozyme, which are helpful for understanding the prevalence of diseases behind the eye or in other parts of the body. For instance, the detection of glucose in tears can be used to detect eye diseases (e.g., glaucoma, dry eyes, macular edema, keratitis, corneal diseases^64^, diabetic retinopathy, and diabetic macular edema^65,66^) in ophthalmology. The rates of blindness and retinopathy in individuals with high blood glucose are 25 times and 21–36% higher than those in healthy individuals, respectively^67^. Therefore, it is of critical importance to continuously and efficiently detect the glucose concentration in tears for early medical intervention. By immobilizing glucose oxidase on the surface of a PDMS contact lens, a soft contact lens (SCL) biosensor can be used to correlate the charge transfer from the oxidation–reduction reaction (i.e., glucose oxidized to gluconic acid) to the glucose concentration in tears (Fig. 5a)^64^. The SCL biosensor has a range of detection of 0.03–5.0 mmol L^−1^ to include the normal tear glucose level (0.14 mmol L^−1^). Although a high water content is desirable to increase oxygen transmission in the lens, it also results in a more fragile lens, which presents challenges in removal, insertion, and cleaning. A fluorescent, glucose-sensitive silicone hydrogel (SiHG) contact lens based on a glucose-sensitive fluorophore (Glu-SF) has been introduced to maintain rapid fluid transfer and high oxygen permeability while allowing the remote optical detection of tear glucose to avoid direct sensor contact with the eye (Fig. 5b)^7^. The interpenetrating polymer network in SiHG contact lenses also provides a reversible glucose response and long-term Glu-SF stability^7^. Water channels in the lens allow rapid tear transport, whereas silicone-rich areas enable continuous and high oxygen transport. An optical glucose sensor was demonstrated by imprinting holographic diffusers on a glucose-sensitive bis-boronic acid-functionalized hydrogel. The diffusion spectrum of the glucose sensor is determined by the lattice size and periodicity of holographic diffusers. In addition to the swelling effect of water, glucose in the solution leads to an increased volume due to anionic boronate−glucose 1:1 complexation. As a result, the diffusion spectrum can be directly correlated to the glucose concentration, which is determined from the measured transition power with a photodiode. Although potential on-body applications have been demonstrated by exploiting the reflection mode, the low sensitivity in low-concentration glucose solutions is not suitable for the measurement of glucose concentrations in tears (Fig. 5c)^68,69^.

The integrated functional contact lens is a highly selective and wireless glucose sensor consisting of a differential enzymatic glucose sensor with a primary sensor and a control sensor, common RE, polyethylene terephthalate substrate, and readout/telecommunication circuit^70^. The signal measured by the primary GOX-based glucose sensor comes from glucose and interference, whereas the control sensor only responds to interference. By taking the difference in the signals from both sensors, the interference can be eliminated. The readout/telecommunication circuit in the system also has a small footprint (0.36 mm^2^), ultralow power consumption (3 ΜW), and fully wireless power/data operation capabilities. Testing and characterization of the integrated functional sensor on a polymer model eye demonstrated good repeatability, molecular interference suppression, and linearity in the range of 0–2 mM glucose to include the normal tear glucose concentration (0.1–0.6 mM). The sensor also demonstrated accurate blood glucose monitoring in the presence of multiple interference factors (Fig. 5d)^70^, and thus has potential to allow further understanding of eye diseases caused by high blood glucose (dry eye, glaucoma, and retinopathy).

A SCL composed of a field-effect transistor-based sensor for glucose sensing, a capacitive pressure sensor for IOP measurement, and an inductive coil for wireless data transmission has been demonstrated recently. By exploiting silver nanowires (AgNWs), graphene, and silicone rubber, all these components have good electrical properties, mechanical softness, and transparency. The glucose concentration and IOP can be obtained wirelessly from the reflection curve using another coil. The change in the resistance of the field-effect transistor due to the absorption of glucose on the graphene channel leads to a change in the reflection magnitude at resonance. IOP increases the radius of curvature of the cornea, thins its dielectric layer and stretches the spiral coil biaxially. As a result, the increase in the capacitance (C) and inductance (L) of the coil leads to a resonance shift (Fig. 5e)^71^.

In addition to biochemical signals, mechanical information, such as the IOP, can also serve as indicators for disease assessment. As one representative example, a black-silicon IOP sensor on an implantable IOL can allow the optical detection of the IOP in fully awake rabbits at a distance of 12 cm away^72^. Furthermore, wearable SCLs can allow continuous monitoring of the IOP for extended periods of time (e.g., 24 h). The sensor exploits an electronic resonant circuit consisting of the resistance of the graphene channel, the inductance of the graphene/AgNW hybrid antenna, the coil and the capacitance of the graphene hybrid source/drain electrode. The increased IOP increases the radius of curvature of the cornea. As a result, the thickness of the dielectric layer is decreased, increasing the capacitance. The lateral expansion of the spiral coil also increases the inductance. The resulting change in the resonance frequency is then related to the IOP. Because of the mechanical deformation-induced measurement, the sensor also exhibits anti-interference performance in the presence of interfering substances (e.g., 50 mM AA, 10 mM lactic acid, and 10 mM UA)^71^.

## Micro/nanodevices for treatment

### Treatment of oral diseases

**Fig. 6:**
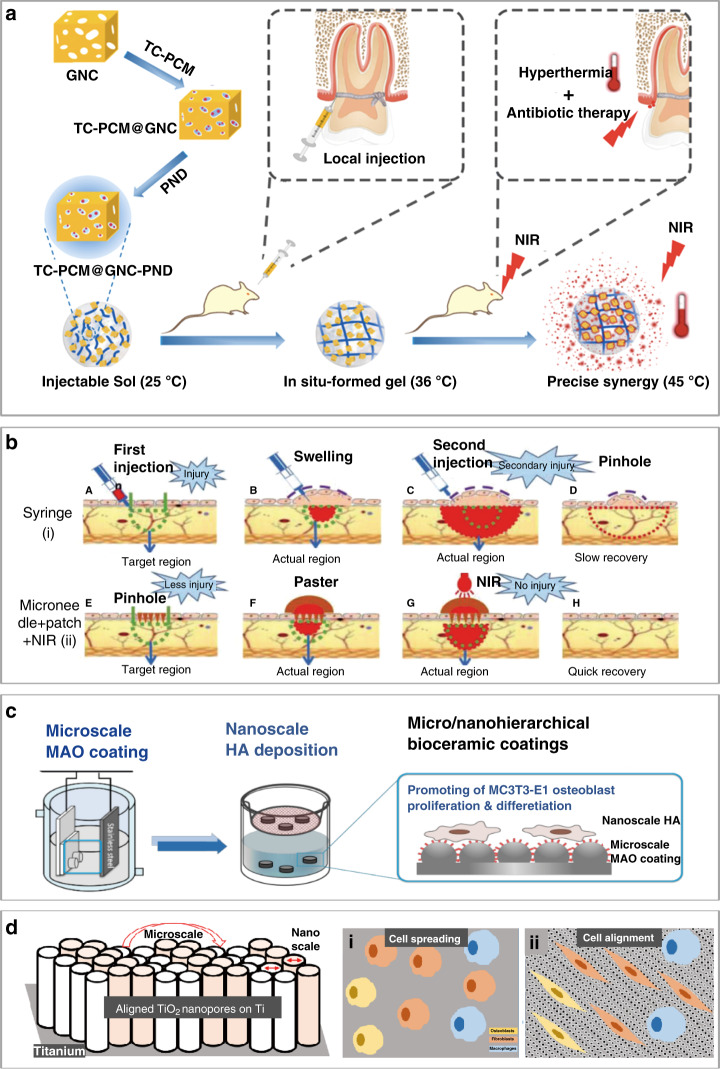
Micro/nanodevices and structures for treatment in stomatology. **a** Schematic showing a nanoantibiotic platform based on TC-PCM@GNC-PND (tetracycline (TC)–phase-change material (PCM) @ gold nanocage (GNC)–poly(N-isopropylacrylamide-co-diethylaminoethyl methacrylate) (PND)) with synergistic antibacterial effects^74^. **b** Local drug administration from microneedles combined with NIR for better and further target penetration, resulting in little damage, no swelling, and fast recovery^78^. **c** Nano- and microstructures with nanoscale hydroxyapatite (Ca_10_(PO_4_)_6_(OH)_2_, HA) prepared by the microarc oxidation (MAO) technique for improved osteoconductivity/osteoinductivity and increased protein adsorption, angiogenesis, and bone bonding^81^. **d** Aligned TiO_2_ nanopores on a Ti substrate with preserved roughness to selectively regulate the proliferation and arrangement of specific cells. Top view schematic of spreading of various cells (i.e., osteoblasts, fibroblasts, and macrophages) on (i) bare Ti and (ii) aligned TiO_2_ nanopores^18^

Local administration and surgical treatment are often used for periodontitis, oral cancer (common in people aged 40–60 years who smoke and drink alcohol), and oral mucosal (ulcer) diseases^73^. Caused by the bacterial infection of periodontal tissue, periodontitis is often treated with mechanical sterilization as the main clinical choice. However, it is difficult to reach deep locations and clean the bacteria attached to the periodontal pocket wall. Therefore, it is helpful to use antibiotic-assisted sterilization for treating periodontitis. However, the effect of treatment is still limited because of undesirable drug leakage. To address this challenge, a light-activable nanoantibiotic platform was developed to accurately deliver antibacterial drugs, such as tetracycline to the diseased area of the tooth upon light and heat exposure (Fig. 6a)^74^. The platform consists of gold nanocages, two thermosensitive gatekeepers, phase-change materials, and the thermosensitive polymer poly(N-isopropylacrylamide-co-diethylaminoethyl methacrylate).

Although radiotherapy, chemotherapy, and surgical treatment are often used after the diagnosis of oral cancer, minimally invasive approaches for drug delivery and pain relief are still the first choice. As a potential solution to poor drug delivery and leakage, chemotherapy drugs have been combined with a microneedle patch as an efficient and minimally invasive method for local drug delivery to treat oral cancer^75^. Based on the fluorescence signal of DOX in a 3D tissue model, drug diffusion from DOX-coated PLGA microneedles placed on the buccal mucosa in a pig model was evaluated. DOX was evenly distributed up to a depth of 3 mm in the cheek tissue of the cadaver, indicating drug release beyond the surface into the tumor and the potential for treating oral cancer^15^.

Compared with existing drug preparation methods for oral mucosal diseases (e.g., spray, paste, diaphragm, gel, and patch)^76^ or even mucosal injection alone, mucosal injection with electroporation is more effective in overcoming the epithelial barrier for drug and mucosal vaccine delivery to increase systemic IgG and mucosal IgA Ab responses^77^. Microneedle patches with electroporation with different geometric designs for (trans)mucosal drug delivery have also been evaluated. Needles with a high aspect ratio result in a fluorescence signal deep in the skin, whereas needles with the sharpest tip and a pitch angle of 15° result in the largest fluorescent area^77^, providing a new tool for clinical applications. The microneedles can also be combined with a sticky hydrogel patch and heat to form a new system for the painless transdermal administration of anesthesia. Even without adhesiveness, the system can achieve good adhesion with the oral mucosa to avoid the leakage of anesthetic drugs. After loading with AuNPs, further penetration of the anesthetic drugs can be achieved with NIR light (Fig. 6b)^78^. Easy-to-shape hydrogel patches can also be conveniently adjusted for individual needs in clinical applications.

Missing teeth are usually replaced by oral implants^79^. In addition to treatment devices, micro/nanostructures can also help enhance the function of oral implants because natural bone tissues have a micro/nanoscale hierarchical structure with a mineral phase composed of calcium and phosphorus^80^. Therefore, oral implants with surface modifications promote the proliferation and osteogenic differentiation of osteoblasts for osseointegration in bone repair^81^. The microscale ceramic coating on a titanium (Ti) substrate in one study was created by a microarc oxidation technique, which provides high hardness and wear resistance, as well as phosphoric acid to form a bioactive calcium phase on the implant surface for a hard tissue interface (Fig. 6c)^81^. Because hydroxyapatite is the main inorganic component in mammalian bones and teeth, nanoscale hydroxyapatite also promotes the adhesion of osteoblastic cells.

Furthermore, improvement in the bioactivity of conventional bone/dental implants can be achieved by nanoengineered Ti to selectively reduce the proliferation of macrophages (immunomodulation), while augmenting the activity of osteoblasts (osseointegration) and fibroblasts (soft tissue integration)^18^. After the generation of dual micro- and nanorough, horizontally aligned TiO_2_ nanopores on the surface of microgrooved Ti, bone, and dental implants can selectively regulate the proliferation and arrangement of specific cells (Fig. 6d)^18^. The surface properties of dental implants can also influence biological seal formation and bone tissue integration between the implant and the gingival soft tissue^14^. In general, the diameter of the implant is selected to be slightly larger than the diameter of the prepared implant site, resulting in the press-fit effect. After implantation in the alveolar bone, the press-fit effect leads to growth from the alveolar bone wall toward the center of the implant, resulting in osseointegration between the bone and the implant. Patterns of microgrooves with different widths and TiO_2_ nanotubes have also been prepared on Ti substrate surfaces for dental implant designs^82,83^. Different from carbon nanotubes with gaps that tend to lead to deformation and breakage, the nanopore structure is compact and dense for enhanced mechanical strength. Aligned TiO_2_ nanopores on a Ti substrate with preserved roughness selectively regulate the proliferation and arrangement of specific cells (i.e., osteoblasts, fibroblasts, and macrophages; Fig. 6d(i), (ii))^18^.

### Treatment of eye diseases

**Fig. 7:**
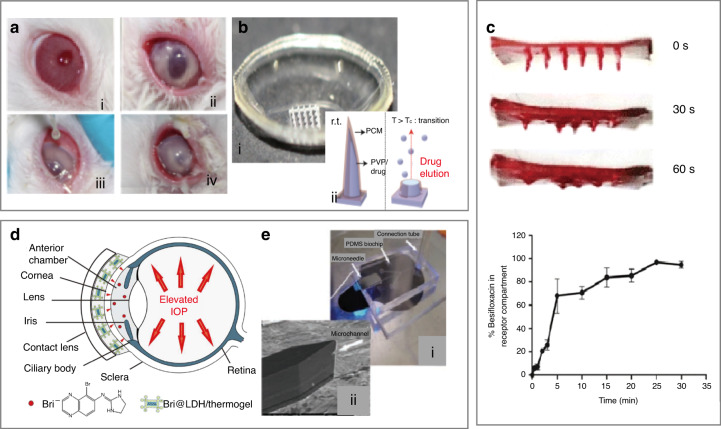
Drug delivery devices for ocular diseases. **a** Images of rabbit eyes before and 24 h after inoculation within the matrix: condition of rabbit eyes (I) before infection, (II) 24 h after inoculation, (III) during administration, and (IV) 24 h after administration^5^. **b** (i) Optical image of the mold for the fabrication of a soluble microneedle eye mask and (ii) schematic diagram of the mechanism of drug delivery by soluble microneedles^5,6^. **c** Besifloxacin observed at 0, 30, and 60 s during microneedle dissolution. Release of besifloxacin loaded on microneedles^4^. **d** Schematic showing a novel layered double hydroxide nanoparticle/thermogel composite drug delivery system (DDS) for the treatment of severe glaucoma^86^. **e** (i) Microneedles integrated with the PDMS microfluidic chip and (ii) the microchannel tip entry^90^

As the most common type of eye disease, an eye infection caused by fungal keratitis or RP^84^ can affect vision or cause blindness (the second leading cause of blindness in developing countries). Eye infection may also cause iris neovascularization in patients with neovascular glaucoma^28^. Therefore, the timely treatment of eye infections is necessary to avoid secondary diseases. New therapeutic agents and effective administration methods have been investigated to achieve the desired concentration of therapeutic agents in target tissues after ocular exposure to *Candida albicans* in vivo (Fig. 7a)^5^. Composed of polyvinyl pyrrolidone and polyvinyl alcohol, the polymer microneedle quickly dissolves within 5 min after being applied to the cornea (water content >81.4%) to deliver the anti-inflammatory drug besifloxacin for the treatment of eye infection (Fig. 7b)^5,6^. Because the microneedle patch loaded with besifloxacin can completely dissolve within 60 s (ref. ^4^), the retention percentage of besifloxacin in the cornea increases linearly within the first 5 min, followed by a slower increase to reach 100% in 25 min (Fig. 7c)^4^. Biodegradable microneedles can also be used to deliver methotrexate into the deep scleral pouch in rabbits for treating primary vitreoretinal lymphoma^85^. By effectively maintaining the level of methotrexate in the eye, the delivery of methotrexate (10% wt) with rapidly dissolving microneedles avoids the side effects (e.g., endophthalmitis, cataract, and vitreous hemorrhage) of the multiple injections commonly required for the intravitreal injection (IVS) of methotrexate. Microneedles, including hollow microneedles, soluble microneedles, and degradable microneedles, are widely used in drug delivery. In addition, mesoporous particles and hydrogels can achieve sustained drug release in ocular drug delivery^86–88^. For severe glaucoma, a drug delivery system based on a layered double hydroxide nanoparticle/thermogel composite can effectively relieve the IOP for at least 7 days. The sustained drug release profile is attributed to the slow diffusion of brimonidine in the thermogel matrix, and the shield coating around the nanoparticles due to the interactions between the copolymer and nanoparticles (Fig. 7d)^86^.

Combined with a PDMS microfluidic chip, microneedles with side openings can allow drug delivery to the CNS for indirect intranasal treatment^89^. For instance, the intranasal delivery of erythropoietin into mice with retinal remodeling can effectively alleviate the morphological destruction induced by N-methyl-N-nitrosourea (Fig. 7e)^90^. Compared with intravenous delivery, intranasal delivery allows the regulation of retinal degenerative cell apoptosis and inhibition of oxidation for more effective protection and erythropoiesis, which also reduces ocular tissue damage^91^. Moreover, drug innovations have greatly improved the therapeutic efficacy. Because hydrogen can selectively neutralize cytotoxic reactive oxygen species and reduce harmful inflammation, local instillation, or IVS has been used to treat local injuries and chronic inflammation^92^. As an ocular anti-inflammatory drug, hydrogen can also significantly reduce the circulating levels of a variety of proinflammatory cytokines (e.g., IL-1β, IL-6, ligand 2 (CCL2), and tumor necrosis factor-α) and ameliorate the accumulation of activated microglia^93^. After being supplied to the subject in a direct manner through a ventilator circuit, mask, or nasal cannula, hydrogen can penetrate membranes and diffuse to organelles (e.g., mitochondria and nuclear DNA).

Different from existing wrinkle treatment methods (e.g., surgery, chemical peeling, botulinum toxin (BONT) injection, filler injection, and laser-based methods)^94,95^, microneedles have recently attracted increasing attention. In one example, the injection of BONT/A with a microneedle fractional radiofrequency device improved static and dynamic wrinkles, demonstrating prospects for periorbital regeneration^96^. As two representative hydrophilic antiwrinkle compounds, AA is a well-known alternative vitamin with antioxidant properties, whereas retinyl retinoate (RR) is an oil-soluble retinol derivative with antiwrinkle activity. Significant wrinkle improvement was observed after the application of a dissolving microneedle patch loaded with AA or RR (AA- or RR-DMN patch) for 12 weeks^97^. Higher levels of the neurotransmitter acetylcholine in the human orbit lead to more severe wrinkles, so the small peptide acetyl hexapeptide 3 (AHP-3), which can inhibit the release of acetylcholine, effectively reduces the formation of wrinkles^95,98,99^. Transdermal drug delivery by a flexible microneedle patch with an active pharmaceutical ingredient exhibited enhanced penetration of AHP-3 through microporous channels^90,100^. Droplet-borne air-blowing (DAB), a mild (4–25 °C) and rapid (≤10 min) microneedle manufacturing method with high-precision control over the drug content, can be used to produce bilayered DAB microneedles to further enable efficient drug (insulin) delivery without waste (bioavailability up to 96.6 ± 2.4%)^101^. Microneedle acupuncture can be used to create microwounds to induce collagen synthesis, and the microneedle treatment system can stimulate the formation of fibroblasts and new blood vessels in the skin to improve elasticity and reduce facial pores, flushing, and wrinkles^102^.

## Conclusion and future perspective

In this review, we have briefly summarized the current status of micro/nanodevices for the monitoring of oral and ocular diseases and delivery of drugs for treatment. Micro/nanodevices have been demonstrated to automatically collect samples and detect molecular biomarkers, and effectively deliver drugs for disease treatment in vivo at a reduced cost, indicating the potential for extensive clinical translation. While significant strides have been made in the development of micro/nanodevices for disease assessment and treatment, there are still major challenges to be overcome before their practical and clinical applications in stomatology and ophthalmology.

Opportunities exist to extract and detect intracellular proteins and small molecules in the field of oral and eye diseases (e.g., monitoring and analyzing changes in the cellular content and levels of specific proteins in abnormal oral mucosal epithelial cells and corneal cells in vitro). Technologies demonstrated for applications in other diseases can also be applied in oral and eye diseases. For instance, hollow nanoneedles in nanoelectroporation devices can penetrate the cell membrane to enable the intracellular recording of electrical and biochemical signals^103^. Because circulating tumor cells (CTCs) are released from the primary tumor or metastasize through blood vessels into the peripheral circulation, the timely detection of CTCs is important to determine the recurrence and metastasis of malignant tumors, as well as early metastasis in oral cancer. NPM-1 (nucleophosmin), which maintains gene stability and regulates cell apoptosis, is associated with the incidence of oral cancer^104^, and a branched nanostraw (BNS) modified with anti-NPM-1 can capture and detect oral squamous cell carcinoma cells in blood samples extracted from patients in real time. Using a multifunctional BNS with a hierarchical topography modified with a specific biomolecule (anti-EpCAM), CTCs can be specifically and effectively captured, followed by cell perforation, while retaining cell activity with a microfluidic nanoelectroporation system^11,12^.

In the field of oral and eye diseases, micro/nanodevices are still emerging technologies; many are still in the experimental stage, and there is still a certain degree of development required before clinical translation. In some microneedle systems, including glass microneedles, polymer microneedles, metal microneedles, and silicon microneedles, the degree of harm caused to the human body in medical aesthetic applications can reach pass a certain level of efficiency, resulting in performance that is less than ideal. Some flexible sensor devices are used to perform in vitro testing after sampling to filter saliva, one of which is based on the coronavirus. Implementing a wearable, flexible sensor to perform the real-time analysis of secretions, such as throat secretions, will effectively improve the detection efficiency. Currently, detection and treatment are separate, and the realization of micro/nanodevices integrating treatment and detection is a major research direction in the scientific community.

Various components, such as inflammatory substances, of subcutaneous tissue fluid from the skin around the eyes and oral mucosa can be extracted and analyzed with a microneedle patch. As a drug carrier, microneedles can be used for local tumor treatment and simultaneous monitoring by piercing the skin or mucosal stratum corneum of the diseased area. Through the combination of biocompatible hyaluronic acid with nanoparticles made of ethoxypropene-conjugated dextran, biodegradable microneedle patches have been demonstrated to promote the continuous delivery of anti-PD-1 (programmed cell death protein 1) monoclonal Abs for the treatment of oral and maxillofacial melanoma^40,105^. In addition to single drug delivery (e.g., anesthesia, chemotherapy, and vaccine), flexible and personalized microneedle chips with antibacterial properties capable of delivering multiple drugs on demand are also of significant interest for the treatment of local inflammatory or infectious diseases (e.g., oral ulcer and corneal infection). Microneedles and other micro/nanodevices may be applied for the delivery of macromolecular drugs, DNA, and genes for the treatment of genetic diseases. Drug delivery and treatment devices can also be combined with sensing components, especially in stretchable and wearable forms^106–109^, to provide feedback loop control for more effective treatment. Nevertheless, joint efforts from both clinicians and engineers with diverse backgrounds are highly desirable to explore these vast opportunities, and help address the major challenges in this burgeoning field.

## Supplementary information

Supplemental Material

## References

[1] Snyder SB, Ferre RM, Boyd JS (2014). Head and neck sonography in the emergency setting. Ultrasound Clin..

[2] Sorsa T (2011). Collagenase-2 (MMP-8) as a point-of-care biomarker in periodontitis and cardiovascular diseases. Therapeutic response to non-antimicrobial properties of tetracyclines. Pharmacol. Res..

[3] Toker E, Yavuz S, Direskeneli H (2004). Anti-Ro/SSA and anti-La/SSB autoantibodies in the tear fluid of patients with Sjgren’s syndrome. Br. J. Ophthalmol..

[4] Bhatnagar S (2018). Corneal delivery of besifloxacin using rapidly dissolving polymeric microneedles. Drug Deliv. Transl. Res..

[5] Roy G (2019). Amphotericin B containing microneedle ocular patch for effective treatment of fungal keratitis. Int. J. Pharm..

[6] Lee H (2016). A graphene-based electrochemical device with thermoresponsive microneedles for diabetes monitoring and therapy. Nat. Nanotechnol..

[7] Badugu R, Reece EA, Lakowicz JR (2018). Glucose-sensitive silicone hydrogel contact lens toward tear glucose monitoring. J. Biomed. Opt..

[8] Kumar S (2015). Biofunctionalized nanostructured zirconia for biomedical application: a smart approach for oral cancer detection. Adv. Sci..

[9] Chen L (2020). Detection of 2019-nCoV in saliva and characterization of oral symptoms in COVID-19 patients. Cell Prolif..

[10] Chang L (2016). Controllable large-scale transfection of primary mammalian cardiomyocytes on a nanochannel array platform. Small.

[11] He G (2019). Multifunctional branched nanostraw-electroporation platform for intracellular regulation and monitoring of circulating tumor cells. Nano Lett..

[12] He G (2018). Hollow nanoneedle-electroporation system to extract intracellular protein repetitively and nondestructively. ACS Sens.

[13] Park S (2016). Intracellular delivery of molecules using microfabricated nanoneedle arrays. Biomed. Microdevices.

[14] Wang M, Hu L, Xu C (2017). Recent advances in the design of polymeric microneedles for transdermal drug delivery and biosensing. Lab Chip.

[15] Ma Y, Boese SE, Luo Z, Nitin N, Gill HS (2015). Drug coated microneedles for minimally-invasive treatment of oral carcinomas: development and in vitro evaluation. Biomed. Microdevices.

[16] Yu J (2015). Microneedle-array patches loaded with hypoxia-sensitive vesicles provide fast glucose-responsive insulin delivery. Proc. Natl Acad. Sci. USA.

[17] Huang D (2018). Efficient delivery of nucleic acid molecules into skin by combined use of microneedle roller and flexible interdigitated electroporation array. Theranostics.

[18] Gulati K, Moon HJ, Li T, Sudheesh Kumar PT, Ivanovski S (2018). Titania nanopores with dual micro-/nano-topography for selective cellular bioactivity. Mater. Sci. Eng. C Mater. Biol. Appl..

[19] Samei, E. et al. Three-dimensional metal artifact reduction method for dental conebeam CT scanners. In *Medical Imaging 2009: Physics of Medical Imaging*, Vol. 7258, 114 (SPIE, Florida, 2009).

[20] Villiger M (2018). Evaluation and review of body fluids saliva, sweat and tear compared to biochemical hydration assessment markers within blood and urine. Eur. J. Clin. Nutr..

[21] Kweon MN (2011). Sublingual mucosa: a new vaccination route for systemic and mucosal immunity. Cytokine.

[22] Baldini C (2011). Proteomic analysis of saliva a unique tool to distinguish primary Sjögren’s syndrome from secondary Sjögren’s syndrome and other sicca syndromes. Arthritis Res. Ther..

[23] Aydin S (2007). A comparison of ghrelin, glucose, alpha-amylase and protein levels in saliva from diabetics. J. Biochem. Mol. Biol..

[24] Lockwood D, Einstein DM, Davros WJ (2006). Diagnostic imaging radiation dose and patients’ concerns. Clevel. Clin. J. Med..

[25] Taylor JJ, Preshaw PM (2016). Gingival crevicular fluid and saliva[J]. Periodontology.

[26] Malathi L, Masthan KMK, Balachander N, Babu NA, Rajesh E (2013). Estimation of salivary amylase in diabetic patients and saliva as a diagnostic tool inearly diabetic patients. J. Clin. Diagn. Res..

[27] Song HK, Noh EM, Kim JM, You YO, Lee YR (2019). Reversine inhibits MMP-3, IL-6 and IL-8 expression through suppression of ROS and JNK/AP-1 activation in interleukin-1-stimulated human gingival fibroblasts. Arch. Oral. Biol..

[28] Hou XR, Miao H, Tao Y, Li XX, Wong IY (2015). Expression of cytokines on the iris of patients with neovascular glaucoma. Acta Ophthalmol..

[29] Wu X, Tao Y, Qiu Q, Wu X (2018). Application of image recognition-based automatic hyphae detection in fungal keratitis. Australas. Phys. Eng. Sci. Med..

[30] Guell JL (2008). Five-year follow-up of 399 phakic Artisan-Verisyse implantation for myopia, hyperopia, and/or astigmatism. Ophthalmology.

[31] David, A. L. & Eve, J. H. Glaucoma and its treatment: a review. *Am. J. Health Syst. Pharm.* **62**, 691–699 (2005). 10.1093/ajhp/62.7.691 15790795

[32] Kingman S (2004). Glaucoma is second leading cause of blindness globally. Bull. World Health Organ..

[33] Malone JD, El-Haddad MT, Yerramreddy SS, Oguz I, Tao YK (2019). Handheld spectrally encoded coherence tomography and reflectometry for motion-corrected ophthalmic optical coherence tomography and optical coherence tomography angiography. Neurophotonics.

[34] Rentka A (2017). Evaluation of commonly used tear sampling methods and their relevance in subsequent biochemical analysis. Ann. Clin. Biochem.

[35] Hartman RR, Kompella UB (2018). Intravitreal, subretinal, and suprachoroidal injections: evolution of microneedles for drug delivery. J. Ocul. Pharm. Ther..

[36] Grus FH, Podust VN, Kai B, Lackner K, Pfeiffer N (2005). SELDI-TOF-MS ProteinChip array profiling of tears from patients with dry eye. Investig. Ophthalmol. Vis. Sci..

[37] Zhao X, Ramsey KE, Stephan DA, Russell P, Factor G (2004). Gene and protein expression changes in human trabecular meshwork cells treated with transforming growth factor-beta. Investig. Ophthalmol. Vis. Sci..

[38] Bhattacharya SK (2006). Proteomics implicates peptidyl arginine deiminase 2 and optic nerve citrullination in glaucoma pathogenesis. Investig. Ophthalmol. Vis. Sci..

[39] Bai W (2018). Patchable micro/nanodevices interacting with skin. Biosens. Bioelectron..

[40] Ye Y, Yu J, Wen D, Kahkoska AR, Gu Z (2018). Polymeric microneedles for transdermal protein delivery. Adv. Drug Deliv. Rev..

[41] Jeerapan I, Sempionatto JR, Pavinatto A, You JM, Wang J (2016). Stretchable biofuel cells as wearable textile-based self-powered sensors. J. Mater. Chem. A Mater..

[42] kim J, Imani S, Araujo WRD, Warchall J, Valdes C (2015). Wearable salivary uric acid mouthguard biosensor with integrated wireless electronics. Biosens. Bioelectron..

[43] Moyer J, Wilson D, Finkelshtein I, Wong B, Potts R (2012). Correlation between sweat glucose and blood glucose in subjects with diabetes. Diabetes Technol. Ther..

[44] Yang X, Cheng H (2020). Recent developments of flexible and stretchable electrochemical biosensors. Micromachines.

[45] Zhang Y (2020). Skin-interfaced microfluidic devices with one-opening chambers and hydrophobic valves for sweat collection and analysis. Lab Chip.

[46] Chang L (2016). Micro-/nanoscale electroporation. Lab Chip.

[47] Dong Z (2020). On-chip multiplexed single-cell patterning and controllable intracellular delivery. Microsyst. Nanoeng.

[48] Chen XJ, Zhang XQ, Liu Q, Zhang J, Zhou G (2018). Nanotechnology: a promising method for oral cancer detection and diagnosis. J. Nanobiotechnol..

[49] Weigum SE (2010). Nano-bio-chip sensor platform for examination of oral exfoliative cytology. Cancer Prev. Res..

[50] Xu H (2020). High expression of ACE2 receptor of 2019-nCoV on the epithelial cells of oral mucosa. Int. J. Oral. Sci..

[51] Liu Y (2012). Targeted imaging of activated caspase-3 in the central nervous system by a dual functional nano-device. J. Control Release.

[52] Jia W (2013). Electrochemical tattoo biosensors for real-time noninvasive lactate monitoring in human perspiration. Anal. Chem..

[53] Skaria E, Patel BA, Flint MS, Ng KW (2019). Poly(lactic acid) carbon nanotube composite microneedle arrays for dermal biosensing. Anal. Chem..

[54] Arakawa T (2020). A wearable cellulose acetate-coated mouthguard biosensor for in vivo salivary glucose measurement. Anal. Chem..

[55] Almusawi MA, Gosadi I, Abidia R, Almasawi M, Khan HA (2018). Potential risk factors for dental caries in Type 2 diabetic patients. Int. J. Dent. Hyg..

[56] Ji X (2016). Highly sensitive metabolite biosensor based on organic electrochemical transistor integrated with microfluidic channel and poly(N-vinyl-2-pyrrolidone)-capped platinum nanoparticles. Adv. Mater. Technol..

[57] Macri E (2014). Atherogenic cholesterol-rich diet and periodontal disease. Arch. Oral. Biol..

[58] Eom KS (2020). Sensitive and non-invasive cholesterol determination in saliva via optimization of enzyme loading and platinum nano-cluster composition. Analyst.

[59] Lestrell E, Patolsky F, Voelcker NH, Elnathan R (2020). Engineered nano-bio interfaces for intracellular delivery and sampling: applications, agency and artefacts. Mater. Today.

[60] Yeh PT, Casey R, Glasgow BJ (2013). A novel fluorescent lipid probe for dry eye: retrieval by tear lipocalin in humans. Investig. Ophthalmol. Vis. Sci..

[61] Shin MK (2020). Matrix metalloproteinase 9-activatable peptide-conjugated hydrogel-based fluorogenic intraocular-lens sensor. Biosens. Bioelectron..

[62] Hao XD (2017). De novo mutations of TUBA3D are associated with keratoconus. Sci. Rep..

[63] Abdalkader R, Kamei KI (2020). Multi-corneal barrier-on-a-chip to recapitulate eye blinking shear stress forces. Lab Chip.

[64] Chu M (2011). Biomedical soft contact-lens sensor for in situ ocular biomonitoring of tear contents. Biomed. Microdevices.

[65] Gilbert C, Gordon I, Mukherjee CR, Govindhari V (2020). Guidelines for the prevention and management of diabetic retinopathy and diabetic eye disease in India: a synopsis. Indian J. Ophthalmol..

[66] Lyu Y, Zeng X, Li F, Zhao S (2019). The effect of the duration of diabetes on dry eye and corneal nerves. Cont. Lens Anterior Eye.

[67] Gebhart S (2003). Glucose sensing in transdermal body fluid collected under continuous vacuum pressure via micropores in the stratum corneum. Diabetes Technol. Ther..

[68] Elsherif M, Hassan MU, Yetisen AK, Butt H (2018). Glucose sensing with phenylboronic acid functionalized hydrogel-based optical diffusers. ACS Nano.

[69] Elsherif M, Hassan MU, Yetisen AK, Butt H (2018). Wearable contact lens biosensors for continuous glucose monitoring using smartphones. ACS Nano.

[70] Yao H (2012). A contact lens with integrated telecommunication circuit and sensors for wireless and continuous tear glucose monitoring. J. Micromech. Microeng..

[71] Kim J (2017). Wearable smart sensor systems integrated on soft contact lenses for wireless ocular diagnostics. Nat. Commun..

[72] Lee JO (2017). Biocompatible multifunctional black-silicon for implantable intraocular sensor. Adv. Healthc. Mater.

[73] Arafa MG, Ghalwash D, El-Kersh DM, Elmazar MM (2018). Propolis-based niosomes as oromuco-adhesive films: a randomized clinical trial of a therapeutic drug delivery platform for the treatment of oral recurrent aphthous ulcers. Sci. Rep..

[74] Zhang L (2020). Light-activable on-demand release of nano-antibiotic platforms for precise synergy of thermochemotherapy on periodontitis. ACS Appl Mater. Interfaces.

[75] Jain RA (2000). The manufacturing techniques of various drug loaded biodegradable poly(lactide-co-glycolide) (PLGA) devices. Biomaterials.

[76] Proniuk S, Dixon SE, Blanchard J (2001). Investigation of the utility of an in vitro release test for optimizing semisolid dosage forms. Pharm. Dev. Technol..

[77] Creighton RL, Woodrow KA (2019). Microneedle-mediated vaccine delivery to the oral mucosa. Adv. Health. Mater..

[78] Zhang F (2019). Microneedles combined with a sticky and heatable hydrogel for local painless anesthesia. Biomater. Sci..

[79] Liu T, Mu Z, Yu T, Wang C, Huang Y (2019). Biomechanical comparison of implant inclinations and load times with the all-on-4 treatment concept: a three-dimensional finite element analysis. Comput. Methods Biomech. Biomed. Engin..

[80] Zhou P (2019). Screening the optimal hierarchical micro/nano pattern design for the neck and body surface of titanium implants. Colloids Surf. B Biointerfaces.

[81] Chen K-C, Lee T-M, Kuo N-W, Liu C, Huang C-L (2020). Nano/micro hierarchical bioceramic coatings for bone implant surface treatments. Materials.

[82] Zhou P (2020). Controlling cell viability and bacterial attachment through fabricating extracellular matrix-like micro/nanostructured surface on titanium implant. Biomed. Mater..

[83] Li Z (2017). TiO2 nanorod arrays modified Ti substrates promote the adhesion, proliferation and osteogenic differentiation of human periodontal ligament stem cells. Mater. Sci. Eng. C Mater. Biol. Appl..

[84] Tao Y (2019). CoPP-Induced-Induced HO-1 overexpression alleviates photoreceptor degeneration with rapid dynamics: a therapeutic molecular against retinopathy. Investig. Opthalmol. Vis. Sci..

[85] Palakurthi NK, Correa ZM, Augsburger JJ, Banerjee RK (2011). Toxicity of a biodegradable microneedle implant loaded with methotrexate as a sustained release device in normal rabbit eye: a pilot study. J. Ocul. Pharm. Ther..

[86] Sun J (2017). Sustained release of brimonidine from a new composite drug delivery system for treatment of glaucoma. ACS Appl Mater. Interfaces.

[87] Hu C (2018). Local delivery and sustained-release of nitric oxide donor loaded in mesoporous silica particles for efficient treatment of primary open-angle glaucoma. Adv. Health. Mater..

[88] Yang H, Tyagi P, Kadam RS, Holden CA, Kompella UB (2012). Hybrid dendrimer hydrogel/PLGA nanoparticle platform sustains drug delivery for one week and antiglaucomaeffects for fourdays following one-time topical administration. ACS Nano.

[89] Peng YJ, Kau YC, Wen CW, Liu KS, Liu SJ (2010). Solvent-free biodegradable scleral plugs providing sustained release of vancomycin, amikacin, and dexamethasone-an in vivo study. J. Biomed. Mater. Res. A.

[90] Paik S-J (2004). In-plane single-crystal-silicon microneedles for minimally invasive microfluid systems. Sens. Actuators A Phys..

[91] Tao Y (2019). Intranasal administration of erythropoietin rescues the photoreceptors in degenerative retina: a noninvasive method to deliver drugs to the eye. Drug Deliv..

[92] Shim W (2019). Catechin solubilization by spontaneous hydrogen bonding with poly(ethylene glycol) for dry eye therapeutics. J. Control Release.

[93] Tao Y (2016). The potential utilizations of hydrogen as a promising therapeutic strategy against ocular diseases. Ther. Clin. Risk Manag..

[94] Aust MC, Fernandes D, Kolokythas P, Kaplan HM, Vogt PM (2008). Percutaneous collagen induction therapy: an alternative treatment for scars, wrinkles, and skin laxity. Plast. Reconstr. Surg..

[95] Lim SH (2020). Geometrical optimisation of a personalised microneedle eye patch for transdermal delivery of anti-wrinkle small peptide. Biofabrication.

[96] Jeon IK, Chang SE, Park GH, Roh MR (2013). Comparison of microneedle fractional radiofrequency therapy with intradermal botulinum toxin a injection for periorbital rejuvenation. Dermatology.

[97] Kim M, Yang H, Kim H, Jung H, Jung H (2014). Novel cosmetic patches for wrinkle improvement: retinyl retinoate- and ascorbic acid-loaded dissolving microneedles. Int. J. Cosmet. Sci..

[98] Yamauchi PS, Lowe NJ (2004). Botulinum toxin types A and B: comparison of efficacy, duration, and dose-ranging studies for the treatment of facial rhytides and hyperhidrosis. Clin. Dermatol..

[99] Lim SH, Sun Y, Madanagopal TT, Kang VRL (2018). Enhanced skin permeation of anti-wrinkle peptides via molecular modification. Sci. Rep..

[100] Donnelly RF, Singh Raj, T. R, Woolfson AD (2010). Microneedle-based drug delivery systems: microfabrication, drug delivery, and safety. Drug Deliv..

[101] Kim JD, Kim M, Yang H, Lee K, Jung H (2013). Droplet-born air blowing: novel dissolving microneedle fabrication. J. Control Release.

[102] Cho JH (2015). Effects of Jae-Seng acupuncture treatment on the improvement of nasolabial folds and eye wrinkles. Evid. Based Complement. Altern. Med..

[103] Stewart MP (2016). In vitro and ex vivo strategies for intracellular delivery. Nature.

[104] Peng HH (2020). Upregulated NPM1 is an independent biomarker to predict progression and prognosis of oral squamous cell carcinomas in Taiwan. Head. Neck.

[105] Wang C, Ye Y, Hochu GM, Sadeghifar H, Gu Z (2016). Enhanced cancer immunotherapy by microneedle patch-assisted delivery of anti-PD1 antibody. Nano Lett..

[106] Yi N, Cui H, Zhang LG, Cheng H (2019). Integration of biological systems with electronic-mechanical assemblies. Acta Biomater..

[107] Yi N, Shen M, Erdely D, Cheng H (2020). Stretchable gas sensors for detecting biomarkers from humans and exposed environments. Trends Analyt. Chem.

[108] Zhang L (2020). Wearable circuits sintered at room temperature directly on the skin surface for health monitoring. ACS Appl Mater. Interfaces.

[109] Zhou H (2020). Stretchable piezoelectric energy harvesters and self-powered sensors for wearable and implantable devices. Biosens. Bioelectron..

[110] Takeuchi T (2020). Antibody-Conjugated signaling nanocavities fabricated by dynamic molding for detecting cancers using small extracellular vesicle markers from tears. J. Am. Chem. Soc..

